# Application of a multiplex PCR assay for the detection of gastrointestinal pathogens in a rural African setting

**DOI:** 10.1186/s12879-016-1481-7

**Published:** 2016-04-14

**Authors:** Daniel Eibach, Ralf Krumkamp, Andreas Hahn, Nimako Sarpong, Yaw Adu-Sarkodie, Amelie Leva, Julia Käsmaier, Marcus Panning, Jürgen May, Egbert Tannich

**Affiliations:** Bernhard Nocht Institute for Tropical Medicine (BNITM), Hamburg, Germany; German Center for Infection Research (DZIF), partner site Hamburg-Borstel-Lübeck, Hamburg, Germany; Kumasi Centre for Collaborative Research in Tropical Medicine (KCCR), Kumasi, Ghana; Kwame Nkrumah University of Science and Technology (KNUST), Kumasi, Ghana; Institute for Virology, University Medical Center, Freiburg, Germany

**Keywords:** Ghana, West Africa, Multiplex Polymerase Chain Reaction, Gastrointestinal infections

## Abstract

**Background:**

Despite high morbidity and mortality, the laboratory diagnosis of gastrointestinal infections is largely neglected in tropical African settings. This study aims to apply the Luminex multiplex PCR assay for the diagnosis of gastrointestinal pathogens in rural Ghana to evaluate its usefulness as a routine method.

**Methods:**

A case–control study was conducted at the Agogo Presbyterian Hospital in Ghana. Stool samples were collected from children below 6 years of age with (cases) and without (controls) diarrhoea. Samples were screened for 15 different diarrhoeal pathogens by the Luminex xTAG GPP assay and associations between diarrhoea and gastrointestinal infections and fractions attributable to diarrhea (AF) were determined.

**Results:**

The Luminex PCR assay identified organisms in 96.6 % (*n* = 428) of 443 cases and in 92.5 % (*n* = 221) of 239 selected controls. A mean of 2.5 (standard deviation [SD]: ±1.3) and 2.3 (SD: ±1.3) organisms per sample were detected in cases and controls respectively. An association with diarrhoea was found for rotavirus (adjusted odds ratio [aOR] = 7.2; 95 % confidence interval [CI]: 2.9–18.1), norovirus (aOR = 2.7; 95 % CI: 1.4–5.3) and *Shigella* spp. (aOR = 1.7; 95 % CI: 1.2–2.4) with respective AFs of 12.5 % (95 % CI: 9.6–15.3), 7.9 % (95 % CI: 3.8–11.7) and 16.9 % (95 % CI: 6.9–25.9).

**Conclusion:**

The high proportion of pathogen-positive stool samples with a high number of co-infections in cases and controls suggests a substantial amount of transient or colonizing microorganisms for which treatment is not necessarily implicated. The use of sequential diagnostic algorithms with pathogen specific or quantitative PCRs might be most appropriate for diagnosing gastrointestinal infections.

## Background

Gastrointestinal infections contribute significantly to childhood morbidity and mortality in sub-Saharan Africa [1, 2]. In developing countries the diagnosis of diarrhoeal infections relies mainly on clinical symptoms, which are generally not indicative for a specific pathogen [3, 4]. However, in this region only few laboratories in referral hospitals are able to perform culture methods, enzyme immunoassays, latex agglutination tests or immunochromatography technologies on a regular basis, while laboratories in rural areas are limited to the detection of intestinal parasites via light microscopy [5]. In the absence of diagnostic tests, infections are managed using empirical antibiotic regimens, which are often associated with overuse of broad-spectrum antibiotics and the development of bacterial resistance [6].

In developed countries, laboratories increasingly apply real time PCR assays to diagnose gastrointestinal infections [7, 8]. Multiplex PCR systems have been shown to allow simultaneous, expeditious amplification of several targets with good sensitivity and specificity [3, 9–21]. Nevertheless, the application of multiplex PCRs for the diagnosis of gastrointestinal infections has not yet been evaluated in tropical countries, where a high number of co-infections as well as asymptomatic carriage of bacterial and parasitic pathogens are common among children [22]. Hence, multiplex PCRs might result in a high number of positive samples leading to multiple treatment options instead of narrowing down the diagnosis to the causative pathogen.

Here, the Luminex multiplex PCR assay was applied for the diagnosis of gastrointestinal pathogens in symptomatic and asymptomatic children with the objective to evaluate its usefulness as a routine method in rural African settings.

## Methods

Samples used in the current analysis are a subset of specimens collected in the framework of another study on causes of diarrhoea in Ghanaian children [22]. The case–control study was conducted at the children’s Outpatients Department (OPD) of the Agogo Presbyterian Hospital, a district hospital with 250 beds in the Asante Akim North municipality, Ghana. This municipality has an estimated population of 142,400 inhabitants and is mainly covered by secondary rain forest and cultivated land [23].

Stool samples were collected from children below 6 years of age visiting the hospital’s OPD between June 2007 and October 2008. Patients with watery or bloody diarrhoea, defined as at least three loose stools within the last 24 h, served as cases. During the same period, children attending the OPD without diarrhoeal disease were recruited as study controls. Cases were not matched to controls for analysis.

### Multiplex PCR assay

Immediately after stool collection, the samples were frozen at −20 °C and shipped on dry ice to Germany for further molecular analyses. DNA was extracted using the QIAamp DNA Stool-Kit (Qiagen, Hilden, Germany) and processed with xTAG GPP using a Luminex 200 instrument (Luminex Corporation, Austin, TX) according to the manufacturer’s instructions. PCR targets were adenovirus 41/41, *Clostridium diffcile* toxin A,B, *Cryptosporidium* spp., *Campylobacter* spp., *Entamoeba histolytica,* heat-labile (LT) and heat-stable (ST) toxins of Enterotoxigenic *Escherichia coli* (ETEC), *Escherichia coli* O157, Shiga toxin-producing *Escherichia coli* (STEC), *Giardia lamblia,* norovirus, rotavirus, *Salmonella* spp., *Shigella* spp., *Vibrio cholerae* and *Yersinia enterocolitica.* Identification was based on elevated relative fluorescence above a threshold predetermined by the manufacturer. In case a sample was tested positive for both, *E.coli* O157 and *E.coli* STEC, it was considered as an *E.coli* O157 mono-infection.

### Statistical and epidemiological analyses

Treatment indications were defined according to the NICE (National Institute for Health and Clinical Excellence) guidelines “Diarrhoea and vomiting in children”, which recommend antibiotic treatment for infections with *Giardia lamblia*, *Shigella* spp., *Entamoeba histolytica*, *Vibrio cholera* and *Clostridium difficile*-associated pseudomembranous enterocolitis in children <5 years and with *Salmonella* spp. in children < 6 months of age. For all other pathogens no treatment exists or is not indicated [24].

Categorical variables are reported as frequencies and percentages, whereas continuous variables are reported as means ± standard deviations (SDs) or as medians with interquartile ranges (IQRs). The associations between diarrhoea and gastrointestinal infections were determined by calculating odds ratios (ORs) and 95 % confidence intervals (CIs). Age adjusted ORs (aORs) were calculated via logistic regression using the age groups 0– < 1, 1– < 2 and 2– < 6 year. The attributable fractions (AF) for diarrhoea burden, defined as the proportion of diarrhoea in the study group attributable to a certain pathogen, were calculated using the aORs [25]. To estimate associations in the occurrence of gastrointestinal infections aORs were calculated, indicating whether the presence or absence of a particular organism is affected by a co-infection with another microbe. All data analyses were performed with STATA 12 (StataCorp LP, College Station, USA).

## Results

Between June 2007 and October 2008 443 (65.0 %) cases with diarrhoea and 239 (35.0 %) controls were selected. Cases were, with a median age of 17 months (IQR: 9–32), younger than controls, which had a median age of 19 months (IQR: 12–36). Females were slightly underrepresented in cases (*n* = 194; 43.8 %) and controls (*n* = 106; 44.4 %) (Table 1).

**Table 1:** Composition and demographic characteristics of cases and controls

Characteristic	Cases	Controls
	(*N* = 443)	(*N* = 239)
Sex, female (%)	194 (43.8)	106 (44.4)
Age, median months (IQR)	17 (9–32)	19 (12–36)
Vomiting (%)	205 (46.4)	40 (16.7)
Number of organisms detected (%)		
0	15 (3.4)	18 (7.5)
1	88 (19.9)	46 (19.3)
2	123 (27.8)	75 (31.4)
3	122 (27.5)	56 (23.4)
> 3	95 (21.4)	44 (18.4)

**Fig. 1:**
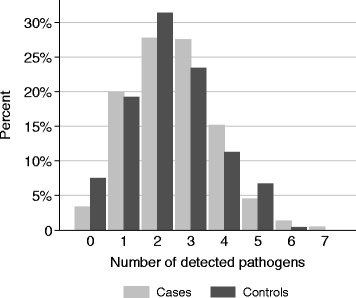
Percentage of patients with number of pathogens detected per stool sample

At least one organism has been identified in 96.6 % (*n* = 428) of the cases and 92.5 % (*n* = 221) of the controls. High numbers of co-infections were detected. Figure 1 presents the proportions of multiple infections diagnosed, showing a comparable distribution of co-infections in cases and controls. On average 2.5 (SD: 1.3) organisms were detected in cases and 2.3 (SD: 1.3) in controls.

The Luminex assay identified 247 (55.8 %) and 144 (60.3 %) enterotoxigenic *Escherichia coli*, 228 (51.5 %) and 125 (52.3 %) *Giardia lamblia,* and 185 (41.8 %) and 76 (31.8 %) *Shigella spp*. as the three most common pathogens in cases and controls, respectively (Table 2). An association with diarrhoea has been found for rotavirus (aOR = 7.2; 95 % CI: 2.9–18.1), norovirus (aOR = 2.7; 95 % CI: 1.4–5.3) and *Shigella* spp. (aOR = 1.7; 95 % CI: 1.2–2.4). The AFs for these infections were 12.5 % (95 % CI: 9.6–15.3) for rotavirus, 7.9 (95 % CI: 3.8–11.7) for norovirus and 16.9 (95 % CI: 6.9–25.9) for *Shigella* spp.

**Table 2:** Crude odds ratio (OR), age-adjusted OR (aOR) on associations between detected pathogens and diarrhoea, sorted by number of cases

Pathogen^a^	Cases (*n* = 443)	Controls (*n* = 239)	Negative PCR-result	Crude OR	aOR
*Escherichia coli* LT/ST (ETEC)	247 (55.8)	144 (60.3)	291	0.8 (0.6–1.2)	0.9 (0.6–1.2)
*Giardia lamblia*	228 (51.5)	125 (52.3)	329	0.9 (0.7–1.3)	1.0 (0.7–1.3)
*Shigella* spp.	185 (41.8)	76 (32.6)	421	1.5 (1.1–2.2)	1.7 (1.2–2.4)
*Campylobacter* spp.	153 (34.5)	78 (32.6)	451	1.1 (0.8–1.5)	1.1 (0.8–1.6)
Rotavirus	64 (14.5)	5 (2.1)	613	7.9 (3.1–25.5)	7.2 (2.9–18.1)
*Escherichia coli* O157	59 (13.3)	37 (15.5)	586	0.7 (0.5–1.2)	0.8 (0.5–1.2)
Norovirus	55 (12.4)	11 (4.5)	616	2.9 (1.5–6.3)	2.7 (1.4–5.3)
*Salmonella* spp.	53 (12.0)	37 (15.5)	592	0.8 (0.5–1.3)	0.8 (0.5–1.3)
*Cryptosporidium* spp.	33 (7.5)	12 (5.0)	637	1.5 (0.7–3.3)	1.4 (0.7–2.9)
Adenovirus (41/41)	17 (3.8)	14 (5.9)	651	0.6 (0.3–1.4)	0.6 (0.3–1.3)
*Escherichia coli* STEC	9 (3.7)	12 (2.7)	661	0.7 (0.3–1.9)	0.7 (0.3–1.8)
*Clostridium diffcile* toxin A,B	2 (0.5)	2 (0.8)	678	-	-
*Entamoaba histolytica*	1 (0.2)	2 (0.8)	679	-	-

^a^ *Vibrio cholerae* and *Yersinia enterocolitica* were not detected in any sample

*OR* odds ratio, *aOR* age adjusted odds ratio

Cases with rotavirus infections are less likely to be simultaneously infected with norovirus (aOR = 0.2; 95 % CI: 0.1–0.7), or *Shigella* spp. (aOR = 0.5; 95 % CI: 0.2–0.9), highlighting that rotavirus cases tend to have no co-infections with another pathogen associated with diarrheal disease. This effect was not observed for simultaneous infections with norovirus and *Shigella* spp. (aOR = 1.1; 95 % CI: 0.6–2.0), while both have a lower association with diarrheal disease.

According to NICE guidelines “Diarrhoea and vomiting in children”, 342 out of 443 (77.2 %) children with diarrhoea should have received antibiotic treatment. Pathogens associated with diarrhoea in this study (i.e., rotavirus, norovirus and *Shigella* spp.) were only detected in 265 (59.8 %) of symptomatic children.

## Discussion

The present study revealed a large proportion (96.6 %) of Luminex positive stool samples among children with diarrhoeal disease, with co-infections detected in more than 75 % of symptomatic cases. The samples analysed in the current study are a subset of samples collected for a larger study on diarrhoeal disease in Ghanaian children. In that study a mix of culture and PCR methods was used to diagnose isolates and a positive result was found in 79.5 % of the analysed samples [22]. A previous multicentre study conducted in sub-Sahara Africa and South Asia detected pathogens in 83 % of diarrhoea samples, using a mixture of conventional bacterial culture methods, immunoassays as well as molecular detection methods for viruses [1]. As expected, lower positivity rates are reported with routine detection methods (i.e. bacterial culture, ELISA, latex agglutination tests, singleplex real-time PCRs, stool microscopy, viral culture) by laboratories from industrialised countries, such as the Netherlands (6.4 %), the United States (8.3 %) and New Zealand (18 %) [9, 17, 19]. The same three laboratories increased the detection frequency of gastrointestinal pathogens 2–4-fold when using a multiplex real-time PCR assay. Compared to the present study, similarly high detection rates (78 %) have been reported from asymptomatic children attending day care facilities in the Netherlands using a quantitative real-time multiplex PCR [26].

Parallel to the high detection rate, co-infections occur in most of the diarrhoeal samples in the present study. Co-infections seem to be less frequent in the United States (14.1 %) and rather exceptional in the Netherlands (0.9 %), although PCR methods were applied in both settings [17, 19], however mainly on adult patients. This difference is probably explained by the high number of asymptomatically circulating pathogens among children living in rural Ghana under low hygiene and sanitary conditions [27]. Those circumstances raise the question, whether highly sensitive multiplex PCR methods are able to provide valuable information for the diagnosis of gastrointestinal infection in sub-Saharan Africa.

Multiplex molecular diagnostic tests might have an important role in epidemiological surveillance, research projects and outbreak investigations [6]. They might also contribute to a better understanding of complex and severe clinical cases as well as to the application of efficient treatment options [21]. However the reduction of antibiotic prescriptions due to multi-parametric molecular diagnostic tools is still debated. Previous studies showed increases as well as decreases of antibiotic consumption, when multiplex PCRs for the diagnosis of respiratory pathogens were investigated [28, 29].

The results of this study suggest that around 77 % of symptomatic children in similar settings would have received antibiotic drugs, when treatment decisions are exclusively based on laboratory results. However, the analyses imply that most organisms found by the Luminex assay might be transient or colonizing and probably not causative for the gastrointestinal symptoms. This leads to the assumption that treatment might be oversubscribed when clinicians stringently adhere to the laboratory results. However, some pathogens (e.g. *Giardia lamblia*) cause intermittent symptoms and therefor presently asymptomatic patients might still benefit from treatment. From a public health aspect, treatment of colonizing pathogens might prevent further transmission to vulnerable populations. Hence, epidemiological markers, such as AFs and odds ratios, can only support but not conclude on individual treatment decisions.

Studies from resource-rich settings demonstrated that the use of stool multiplex PCR panels might be cheaper than using different individual methods for the same number of pathogens when all costs of reagents and technicians’ time are taken into account [9, 30]. However those costs might still be too high for rural laboratories in sub-Saharan Africa. Furthermore, the lack of technical expertise on molecular diagnostic techniques might be a constraint.

It might be worth to investigate whether for this study setting a sequential or stepwise diagnostic algorithm might be more suitable and cost-effective. Within such an algorithm, all children with diarrhoea could be tested for pathogens with a high AF in a first screening procedure. Only those children tested negative would undergo further screening for other pathogens. Another approach would be the development of quantitative or semi-quantitative PCR assays, which distinguish between causative and colonizing pathogens [31, 32]. In general, the decision on the most appropriate diagnostic tests for resource-poor settings must not rely on test evaluations in industrialized countries.

The study has several limitations. Sensitivities and specificities of the Luminex PCR assay cannot be assessed since results have not been compared to a reference method or confirmed by another assay. A previous study, which analysed a different sub-population within the same stool sample collection using a mixture of conventional and PCR detection methods, revealed similar AFs, but lower positivity rates [22]. Indeed, the Luminex assay has been evaluated with high sensitivities and specificities in previous studies [18, 19, 21, 30, 33–36]. However the Luminex PCR requires open manipulation of an amplified product, which carries the potential for carryover contamination, resulting in false positive results. We recruited cases as well as controls at the hospital OPD, hence the control group consists of children being sick with conditions other than diarrhoea. Occasionally gastrointestinal pathogens might lead to vomiting without diarrhoeal symptoms, e.g. as described for *Cryptosporidium parvum* [37], which may result in an underestimation of the association between gastrointestinal pathogens and diarrhoea in this study.

## Conclusion

The Luminex xTAG GPP multiplex assay identified a high proportion of stool samples with a high number of co-infections that differed only minimal in case and control groups. These results indicate the presence of a substantial amount of transient or colonizing microorganisms, which are not causative for diarrhoea. In endemic regions, the application of multiplex PCR assays with high sensitivities and specificities might therefore result in potentially unnecessary therapies. Thus, a sequential diagnostic algorithm using pathogen-specific or quantitative PCRs with a priority on pathogens with high AFs (*Shigella* spp., rotavirus, norovirus) might give more clinically relevant results.

### Ethics approval and consent to participate

All participants were informed about the study’s purpose and procedures. Written informed consent was obtained from the parents or the guardian on behalf of the study children prior to study enrolment. Non-participation had no effect on the medical treatment provided. The Committee on Human Research, Publications and Ethics, School of Medical Science, Kwame Nkrumah University of Science and Technology, Kumasi, Ghana, approved the study design and the informed consent procedure.

### Availability of data and materials

All the data will be made available by the corresponding author upon request.

## Abbreviations

AF: attributable fraction
aOR: adjusted odds ratio
CI: confidence interval
ETEC: Enterotoxigenic *Escherichia coli*
IQR: interquartile range
LT: heat-labile toxin
NICE: National Institute for Health and Clinical Excellence
OPD: Outpatients Department
OR: Odds ratio
PCR: polymerase chain reaction
SD: standard deviation
ST: heat-stable toxin
STEC: Shiga toxin-producing E. coli
